# Caveolin-1 overexpression is an early event in the progression of papillary carcinoma of the thyroid

**DOI:** 10.1038/sj.bjc.6600172

**Published:** 2002-03-18

**Authors:** Y Ito, H Yoshida, K Nakano, K Kobayashi, T Yokozawa, K Hirai, F Matsuzuka, N Matsuura, K Kakudo, K Kuma, A Miyauchi

**Affiliations:** Department of Surgery, Kuma Hospital, 8-2-35, Shimoyamate-dori, Chuo-ku, Kobe 650-0011, Japan; Department of Pathology, School of Allied Health Science, Osaka University Faculty of Medicine, 1-7, Yamadaoka, Suita, Osaka 565-0871, Japan; Department of Pathology, Wakayama Medical School, Wakayama, Japan

**Keywords:** caveolin-1, immunohistochemistry, thyroid carcinoma

## Abstract

Caveolin-1 is a major structural component of caveolae, which are plasma membrane microdomains implicated in the regulation of intracellular signalling pathways. Previous *in vitro* and *in vivo* studies on the function of caveolin-1 in carcinoma showed controversial results, indicating that the physiological role of caveolin-1 varies according to the origin of carcinoma. In this study, we investigated caveolin-1 expression in thyroid neoplasms by means of immunohistochemistry using a rabbit polyclonal antibody against caveolin-1. Normal follicular cells did not express caveolin-1. In papillary carcinoma, caveolin-1 expression was observed in high incidence, and especially in microcancer (less than 1.0 cm in diameter), caveolin-1 was positive in all cases except one. In undifferentiated (anaplastic) carcinoma, its incidence was significantly reduced. On the other hand, all cases of follicular carcinoma and adenoma were classified as negative for caveolin-1. These results suggest that caveolin-1 may play a role predominantly in the early phase of papillary carcinoma, whereas it has little influence on follicular tumours.

*British Journal of Cancer* (2002) **86**, 912–916. DOI: 10.1038/sj/bjc/6600172 www.bjcancer.com

© 2002 Cancer Research UK

## 

Caveolin-1 is a 22 kDa protein and a prominent member of the caveolin family. This protein is a major structural component of caveolae, which are the vesicular invaginations of the plasma membrane, and is abundantly present in vascular endothelial cells, adipocytes, smooth muscle cells and fibroblast (Harter and Simons, 1997; Okamoto *et al*, 1998). Previous studies have demonstrated that the caveolin family contains a common domain, a caveolin-scaffolding domain which organises and concentrates sphingolipids and lipid-modified signalling molecules (Song *et al*, 1997a). The function of caveolin-1 has been investigated by previous studies, which showed that caveolin-1 inhibits intracellular signal transduction modulated by various signalling molecules (Li *et al*, 1995; Couet *et al*, 1997; Garcia-Cardena *et al*, 1997; Song *et al*, 1997b; Engelman *et al*, 1998a; Lui *et al*, 1999).

The *caveolin-1* gene is located at human chromosome 7q31.1, and this region is frequently deleted in carcinomas (Engelman *et al*, 1998b). The expression of caveolin-1 protein in carcinomas has also been studied. Thus far, a decreased expression of caveolin-1 has been found in a variety of cell lines such as those of breast carcinoma (Lee *et al*, 1998), lung carcinoma (Racine *et al*, 1999), sarcoma (Wiechen *et al*, 2001), uterine cervical carcinoma (Razani *et al*, 2000), and colon carcinoma (Bender *et al*, 2000). Furthermore, re-expression of caveolin-1 inhibited the growth of breast carcinoma cells (Lee *et al*, 1998) and colony formation of sarcoma cells (Razani *et al*, 2000) and ectopic caveolin-1 expression reduced tumorigenecity of colon carcinoma cells (Bender *et al*, 2000). These results suggest that the *caveolin-1* gene may be a candidate as a tumour suppressor gene as its gene product functions as a negative regulator of tumour progression.

On the other hand, the results of studies for caveolin-1 expression using human carcinoma tissue have been different from those using cell lines. Yang *et al* (1998) showed that the expression of caveolin-1 was elevated in breast and prostate carcinomas and, especially in prostate carcinoma, caveolin-1 expression was more frequently observed in cases with high biological aggressiveness including poor prognosis (Yang *et al*, 1999). Furthermore, caveolin-1 expression was more membranous in ovarian carcinoma with short-term survival (Davidson *et al*, 2001). In colon carcinoma, caveolin-1 expression increased compared to normal epithelium, but it was not linked to the stage (Fine *et al*, 2001). These results indicate that caveolin-1 has some functions other than as a negative regulator of tumour progression, and its physiological role in carcinoma is complicated depending on the origin of the carcinoma and other various circumstances.

Thyroid carcinoma is one of the most common malignancies originating from the endocrine organs. There are two prominent histological types of thyroid carcinoma originating from the follicular cells. One is papillary carcinoma and the other is follicular carcinoma, which is comparatively rare (LiVolsi and Asa, 1994). Generally, the biological character of these carcinomas is mild, but when they dedifferentiate and become anaplastic (undifferentiated), their growing activity is very strong and patients usually have a dire prognosis, in spite of various therapeutic strategies. These polarised characteristics of thyroid carcinoma have prompted many researchers to study the factors linked to the dedifferentiation of papillary and follicular carcinomas. In this study, we investigated caveolin-1 expression in various types of thyroid neoplasm in order to clarify its role, including this point.

## MATERIALS AND METHODS

### Tissue specimens

Tissue specimens of thyroid neoplasms were obtained from 161 patients who underwent surgery in the Department of Surgery, Kuma Hospital. These consisted of 32 anaplastic (undifferentiated) carcinomas, 85 papillary carcinomas, 15 widely invasive and eight minimally invasive follicular carcinomas and 11 follicular adenomas. The patients with anaplastic carcinoma were operated on from 1983 to 2001, and those with widely invasive follicular carcinoma from 1995 to 2001. Other cases were selected from patients who underwent surgery from 1999 to 2001. Of the 85 papillary carcinomas, 23 were microcancers which were less than 1.0 cm in diameter. They were pre-operatively diagnosed or incidentally found in specimens of other diseases such as hyperthyroidism, adenomatous goiter and follicular adenoma. We divided the remaining papillary carcinoma cases into two categories, type A and type B. Type A papillary carcinoma is composed of pure papillary structure, whereas type B carcinoma has any one of the components showing a solid, trabecular or scirrhous growth pattern. For immunohistochemical study, the tissues were fixed with 10% formalin and paraffin-embedded. This project was approved by the ethics committees of the hospital and informed consent was obtained from the participating patients.

### Antibodies

We adopted a rabbit polyclonal antibody against caveolin-1 (N-20) at a dilution of 1:300 as the primary antibody, obtained from Santa Cruz Biotechnology (Santa Cruz, CA, USA).

### Immunohistochemistry

Tissue sections 4 μm thick were dewaxed and endogenous peroxidase activity was blocked with 0.3% hydrogen peroxide in methanol for 15 min. After rinsing in distilled water, the sections were then immersed in 0.03 mol L^−1^ citrate buffer (pH 6.0) and incubated at 95°C for 40 min in a water bath for antigen retrieval. After rinsing in phosphate-buffered saline pH 7.2 (PBS), 10% bovine serum (Wako, Osaka, Japan) was applied for 20 min to block nonspecific reactions. The sections were then incubated with the primary antibody overnight at 4°C. After rinsing in PBS, they were treated with peroxidase-labelled anti-rabbit immunoglobulins (Nichirei, Tokyo, Japan) for 30 min. The peroxidase reaction was visualised by incubating the sections with 0.02% 3,3′-diaminobenzidine tetrahydrochloride in 0.05 M Tris buffer with 0.01% hydrogen peroxide (Nichirei, Tokyo, Japan). The sections were counterstained with haematoxylin. Sections for the negative control were prepared using rabbit immunoglobulins instead of the primary antibody.

### Immunohistochemical evaluation

We regarded cells as immunoreactive when the staining signal was clearly observed in their cytoplasms. We scored immunoreacitivity as follows: (−) less than 10% of cells were immunoreactive; (+) 10–25% of cells were immunoreactive; (++) 25–50% of cells were immunoreactive, and (+++) more than 50% of cells were immunoreactive. The (++) and (+++) cases were considered as positive for caveolin-1.

### Statistical analyses

Chi-square analysis was employed to analyse the relationship between caveolin-1 expression and the pathological classification of thyroid neoplasms. A *P* value of less than 0.05 was considered to be statistically significant.

## RESULTS

**Figure 1:**
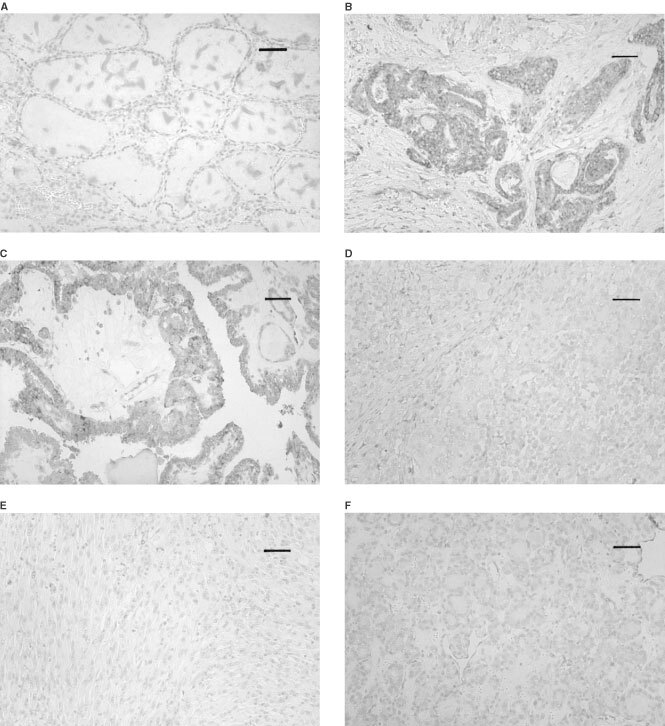
(**A**) Caveolin-1 is negative in normal follicular cells. (**B**) Caveolin-1 expression in microcancer. This case was classified as (+++). (**C**) Caveolin-1 expression in type A papillary carcinoma classified as (++). (**D**,**E**) Caveolin-1 is negative in type B papillary (**D**) and undifferentiated carcinomas (spindle cell type) (**E**). (**F**) Caveolin-1 is negative in this follicular carcinoma, minimally invasive type. Scale bars, 33 μm.

Caveolin-1 immunoreactivity was frequently present in the endothelial cells in blood vessels in the stroma, which were recognised as an internal positive control. Follicular cells of normal thyroid tissue did not express caveolin-1 (Figure 1A

). We then investigated caveolin-1 expression in various types of thyroid neoplasm (Table 1

**Table 1:**
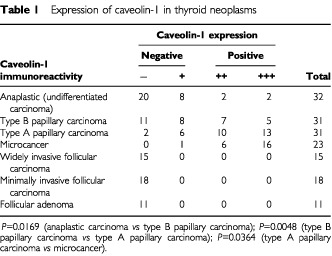
Expression of caveolin-1 in thyroid neoplasms

). Of the 85 papillary carcinomas, 57 cases (67.1%) were judged as positive for caveolin-1. Especially in microcancers, caveolin-1 was positive in all the cases except one (Figure 1B). Of the remaining two types of papillary carcinoma, cases with a pure papillary structure, classified as type A, were more frequently positive than those with other growth patterns (type B) (Figure 1C,D). In anaplastic (undifferentiated) carcinomas, only four cases (12.5%) were positive for caveolin-1, which was significantly lower than in type B papillary carcinomas (Figure 1E).

We also examined caveolin-1 expression in tumours of follicular type, that is, 11 cases of follicular adenoma, 18 cases of minimally invasive follicular carcinoma and 15 cases of widely invasive follicular carcinoma. However, in contrast to the papillary carcinomas, caveolin-1 immunoreactivity was not seen in the tumour cells of these tissues, and all these cases were classified as negative, regardless of histological type (Figure 1F).

## DISCUSSION

In this study, we have demonstrated that caveolin-1 was frequently positive in papillary carcinoma, but not in tumours of the follicular type. In papillary carcinomas, caveolin-1 was more frequently positive in microcancers than those of larger size, indicating that caveolin-1 expression is an early event in papillary carcinoma. An additional more important finding is that caveolin-1 expression significantly decreased in undifferentiated (anaplastic) carcinomas. Anaplastic carcinomas can arise from follicular carcinoma as well as papillary carcinoma, but most are thought to be from papillary carcinoma, because papillary carcinoma is far more common than follicular carcinoma. These results allow us to hypothesise that, in papillary carcinoma, caveolin-1 works as a negative regulator of carcinoma progression and the lack of or decreased expression of this protein is linked to the increase in biological aggressiveness. The reduced expression of caveolin-1 in type B carcinomas compared to type A carcinomas is also reasonable because cases with type B histology were reported to show a poorer prognosis than pure papillary carcinomas (type A), although it is still an open question whether type B cases actually represent dedifferentiation as proposed by Sakamoto *et al* (1983).

The function of caveolin-1 has been intensively investigated by many researchers. Engelman *et al* (1998b) have demonstrated that caveolin-1 negatively regulates the activity of p42/44 MAP kinase, with the result that caveolin-1 dramatically inhibits signalling from EGF-R, Raf, MEK-1 and ERK-2 to the nucleus. Furthermore, similar relationships were observed between caveolin-1 and heterotrimeric G proteins (Li *et al*, 1995), c-Src tyrosine kinase (Song *et al*, 1997b) and nitric oxide synthase (Garcia-Cardena *et al*, 1997), and all these phenomena are mediated through the caveolin-scaffolding domain. Similar results have also been obtained for signal transduction from c-erbB-2 (Engelman *et al*, 1998c) and vascular endothelial growth factor receptor (VEGF-R) (Lui *et al*, 1999). As EGF-R (van der Laan *et al*, 1995), VEGF and VEGF-R (Klein *et al*, 1999) as well as c-erbB-2 (Utrilla *et al*, 1999) are known to be frequently present in papillary carcinoma, these data support the above hypothesis. In contrast, the aberrant expression of c-erbB-2, VEGF, basic FGF and hepatocyte growth factors, as well as inactivation of p53, can, in turn, down-regulate caveolin-1 (Engelman *et al*, 1998c; Lui *et al*, 1999; Razani *et al*, 2000). Furthermore, when EGF-R binds to EGF, it can move out of the caveolae with the modulation of Src kinase (Mineo *et al*, 1999). It is therefore suggested that the abundant expression of various growth factors and the inactivation of tumour suppressor genes, such as p53, may cause the down-regulation or decreased expression of caveolin-1. Regarding the molecular mechanism of down-regulation of caveolin-1, Cui *et al* (2001), have reported hypermethylation of the *caveolin-1* gene promoter in prostate carcinoma and also, mutation of the *caveolin-1* gene has been identified in scirrhous breast carcinomas (Hayashi *et al*, 2001). Further studies of this subject should be carried out on various carcinomas.

On the other hand, caveolin-1 is phosphorylated at tyrosin-14 by c-Src kinase and binds to growth factor receptor-binding protein 7, which results in anchorage-independent growth and EGF-stimulated cell migration (Lee *et al*, 2000). This indicates the possibility that caveolin-1 not only inhibits but also stimulates carcinoma progression and metastasis. In prostate carcinoma, Yang *et al* (1999) have demonstrated that caveolin-1 expression is directly related to the Gleason score, positive surgical margin and lymph node metastasis, and it independently predicts a worse prognosis for patients. They also showed that caveolin-1 can be secreted and can contribute to metastasis in androgen-insensitive prostate carcinoma in an autocrine/paracrine fashion (Tahir *et al*, 2001). It is therefore suggested that the function of caveolin-1 is not uniform in carcinoma, depending on the origin of the carcinoma and on the expression status of various growth factors and hormones. Studies regarding the secreted type of caveolin-1 in thyroid carcinoma will be necessary in the future.

Interestingly, in follicular tumours, no caveolin-1 expression was observed in any cases, regardless of histological type. It is therefore suggested that the regulation of caveolin-1 does not act on or against the progression of follicular tumours. As there is no striking difference between the prognosis of patients with follicular carcinoma and papillary carcinoma, regulation of some other type than caveolin-1 must operate for signal transduction in follicular carcinomas. A previous study observed that c-erbB-2 oncoprotein was expressed in more than 50% of papillary carcinomas but not in follicular carcinomas or undifferentiated carcinomas (Utrilla *et al*, 1999). The difference in the status of these proteins in papillary and follicular carcinomas reflects the difference of entities between these two types of carcinoma.

In summary, this study indicates that, in papillary carcinomas, caveolin-1 plays a role in the early phase and its decreased expression is linked to aggressive characteristics including dedifferentiation. Further studies should be carried out regarding the mechanism of function and/or the lack of function of caveolin-1 in thyroid carcinoma.
